# LncRNA-ENST00000543604 exerts a tumor-promoting effect *via* miRNA 564/AEG-1 or ZNF326/EMT and predicts the prognosis of and chemotherapeutic effect in colorectal cancer

**DOI:** 10.3389/fonc.2022.960481

**Published:** 2022-08-23

**Authors:** Weimin Wang, Zhen Zhou, Xiaojun Dai, Haibo Wang, Jun Jin, Ke Min, Yunfan Wang, Mengying Lv, Yanqing Liu, Yan Zhou

**Affiliations:** ^1^ Institute of Combining Chinese Traditional and Western Medicine, Medical College, Yangzhou University, Jiangsu, China; ^2^ Department of Oncology, Yixing Hospital Affiliated to Medical College of Yangzhou University, Yangzhou University, Jiangsu, China; ^3^ Department of Chinese Medicine, Nanjing University of Chinese Medicine, Jiangsu, Nanjing, China

**Keywords:** ENST00000543604, miRNA 564, AEG-1, ZNF 326, colorectal cancer, chemotherapy

## Abstract

**Objectives:**

Colorectal cancer(CRC) is a common malignant tumor. Recent studies have found that lncRNAs play an important role in the occurrence and development of colorectal cancer.

**Methods:**

Based on high-throughput sequencing results of fresh CRC tissues and adjacent tissues, we identified lncRNA-ENST00000543604 (lncRNA 604) as the research object by qRT-PCR in CRC tissues and cells. We explored the mechanism of lncRNA 604 action by using luciferin reporter, qRT-PCR and Western blot assays. Kaplan-Meier survival analysis and a Cox regression model were used to analyze the correlation of lncRNA 604 and its regulatory molecules with the prognosis of and chemotherapy efficacy in CRC patients.

**Results:**

In this study, we found that the expression levels of lncRNA 604 were increased in CRC. LncRNA 604 could promote CRC cell proliferation and metastasis through the miRNA 564/AEG-1 or ZNF326/EMT signaling axis *in vivo* and *in vitro*. LncRNA 604 could predict the prognosis of CRC and was an independent negative factor. LncRNA 604 exerted a synergistic effect with miRNA 564 or ZNF326 on the prognosis of CRC. LncRNA 604 could improve chemoresistance by increasing the expression of AEG-1, NF-κB, and ERCC1.

**Conclusions:**

Our study demonstrated that lncRNA 604 could promote the progression of CRC *via* the lncRNA 604/miRNA 564/AEG-1/EMT or lncRNA 604/ZNF326/EMT signaling axis. LncRNA 604 could improve chemoresistance by increasing drug resistance protein expression.

## Background

Colorectal cancer is a common malignant tumor in the digestive tract, and its incidence ranks third among malignant tumors worldwide, making it a worldwide health concern (1). The prognosis of CRC mainly depends on early diagnosis and timely surgical treatment: the prognosis is better if the cancer is only confined to the intestinal wall and the prognosis is poor for those patients with extensive cancer invasion and distant metastasis (2). The pathological process of tumor metastasis mainly involves tumor cells spreading from the primary tumor microenvironment to various remote organs and colonizing other sites (3). Despite the popularity of early tumor screening and continuous improvement in treatment, the mortality rate of CRC has decreased in recent years, and metastasis of CRC is still a key factor that causes low clinical efficacy, short survival and poor prognosis (4). Therefore, it is necessary to identify and target oncogenes and tumor suppressor genes involved in the metastasis of CRC to inhibit this process and predict the chemotherapy efficacy and prognosis of CRC patients.

In addition to protein-coding genes that could regulate tumor invasion and metastasis, there are also some noncoding genes involved in this process. Noncoding RNAs mainly include small noncoding RNAs and long-chain noncoding RNAs (lncRNAs). LncRNA refers to a transcript that is longer than 200 nt, lacks an open reading frame, and does not have protein coding functions. LncRNA dysfunction is related to a variety of diseases (5). The abnormal expression of lncRNAs is directly related to the occurrence and development of many malignant tumors (6, 7). With the development of transcriptomics analysis technology, we have found that lncRNAs are related to a series of biological processes, including chromatin modification, transcription interference, alternative splicing, translation initiation, and miRNA decay (8, 9). LncRNAs can interact with proteins, RNAs and lipids and play a key mediating role in the occurrence and development of cancer (10, 11). LncRNAs have become an important part of transcription and epigenetic regulatory networks.

In recent years, differentially expressed lncRNA profiles in CRC tissues and adjacent tissues have been reported. Some lncRNAs, such as lncRNA-ATB and DANCR, could be effective markers for predicting the prognosis of CRC (12, 13). LncRNA-422 and BC032913 can regulate downstream target genes through different mechanisms to affect the proliferation or invasion of CRC cells (14, 15). To identify more lncRNAs in the occurrence and development of CRC, we selected 3 fresh human-derived CRC tissues and corresponding adjacent tissues to detect the expression levels of lncRNAs by high-throughput sequencing. The sequencing results showed that there were 3006 differentially expressed lncRNAs compared with adjacent tissues. According to the copy difference of these lncRNAs that were greater than or equal to 10, the expression of lncRNAs was determined in fresh CRC tissues and adjacent tissues, CRC cells and intestinal mucosal cells. Finally, lncRNA-ENST00000543604 (lncRNA 604) was preliminarily identified as having biological functions through a high-content screening experiment, and the expression of lncRNA 604 was relatively stable in CRC tissues and cells.

In our article, we constructed virus expressing high levels and low levels of lncRNA 604 to systematically study its function. Simultaneously, we used a CRC database tissue chip to study whether lncRNA 604 and its regulatory molecules could predict the prognosis of and chemotherapy efficacy in CRC patients.

## Materials and methods

### Clinical specimens

A total of 470 CRC patients undergoing surgical resection were enrolled from Yixing Hospital affiliated with Yangzhou University Medical College from 2009.01 to 2015.12. All patients provided informed consent. These patients were followed up for at least 5 years, and their pathological diagnosis was confirmed by two pathologists. The clinicopathological characteristics are presented in **Table S1**. The procedures of this study were approved by the Ethics Committee of Yixing Hospital and were performed according to the principles of the Declaration of Helsinki.

### Cell lines and animals

The human CRC cell lines SW620, HCT116, HT29, SW480, RKO, HCT-15 and FHC were purchased from Wuhan Procell Life Science and Technology Co., Ltd. They were cultivated in RPMI-1640 medium supplemented with 10% FBS and 1% penicillin/streptomycin at 37°C and 5% CO_2_.

Four-week-old female BALB/c nude mice were obtained from the Comparative Medicine Laboratory Animal Center [License No. scxk (SU) 2012-0004] of Yangzhou University and maintained under specific pathogen-free conditions. Animal studies were performed in accordance with the National Institutes of Health Guide for the Care and Use of Laboratory Animals.

### Lentiviral infection and generation of stable cell lines

First, we used LV-lncRNA 604-ctrl and LV-lncRNA 604-RNAi-ctrl to infect HCT116 and HT29 cells. Because the lentiviral carried luciferin, we used fluorescein potassium salt to excite the fluorescence, and then observe the infection efficiency through the microscope. HCT116 and HT29 cells were infected with lentivirus (LV)-lncRNA 604, LV-lncRNA 604-RNA interference (RNAi) at a multiplicity of infection (MOI) of 20 and 10 µg/ml polybrene (Shanghai GeneChem Co, Ltd.).

After lentiviral infection for 8 h, these cells were maintained with normal RPMI-1640 medium for 24 h. Subsequently, these cells were incubated in RPMI-1640 with 2 μg/ml puromycin (Gibco-BRL, Gaithersburgh, MD, USA). The knockdown and overexpression efficiency of lncRNA 604 was further analyzed by RT-PCR.

### Construction of a tissue microarray and immunohistochemistry

First, we used HE staining to examine the cancer tissues and corresponding adjacent tissues. Then, we used Shanghai Xinchao Biological Co., Ltd. to make TMAs. These TMAs were stored at -20°C for later use.

The immunostaining protocol was performed according to a previously published article (16). Rabbit monoclonal anti-ZNF326 antibody (1:100, ABclonal Technology, USA) was incubated at 4°C overnight. The staining of ZNF326 in tissue was scored by two pathologists and evaluated by the semiquantitative immunoreactivity score (IRS), as reported elsewhere (17).

### CCK8 assay

HCT116 or HT29 cells (0.5 × 10^3^ cells per well) were seeded in 96-well plates. After treatment, cell growth was detected by Cell Counting Kit-8 (CCK-8) solution (Dojindo Molecular Technology Inc., Shanghai, China) at five time points (24, 48, 72, 96 and 120 h). The absorbance was measured at 450 nm by an automatic microplate reader.

### EdU immunofluorescence assay

HCT116 or HT29 cells were cultured to logarithmic growth. Then, 5×10^3^ cells/well were seeded in a 96-well plate. After 24 h, immunofluorescence analysis was performed according to the protocol of the EdU Kit (RIBOBIO CO, LTD, Guang Zhou, China).

### Cell migration and invasion assay

The upper chambers of a Transwell chamber were coated or not with Matrigel (Millipore, Billerica, MA, USA) with serum-free RPMI 1640 medium at a ratio of 1:2. The cells were resuspended to 5×10^5^ cells/ml, 100 μl cell suspension was added to the upper chamber, and 600 μl RPMI 1640 medium containing 10% FBS was introduced into the lower chamber. After 24 h of incubation, the cells were fixed with 4% formaldehyde for 10 min and stained with 0.1% crystal violet for 5 min. The number of penetrating cells was recorded in five fields of view under an inverted microscope, and the cells were photographed.

### High-content imaging system analysis

HCT116 or HT29 cells were seeded in a 96-well plate at a density of 5 × 10^3^ cells/well and further cultured for 24 h. The 96-well plate was placed into a PerkinElmer Operetta CLS high-content imaging analysis system (PerkinElmer, Waltham, USA). Last, for a 12-h observation, Harmony 4.1 software was used to obtain and analyze the data.

### Fluorescence *in situ* hybridization

The TMA paraffin sections were baked overnight at 65°C. Xylene was deparaffinized twice. The sections were incubated at 100°C for 25 min, digested in pepsin solution for 15 min, and then washed with anhydrous ethanol. Two microliters of probe mixture was dropped on the sections after the above treatment, covered with glass, and sealed with rubber glue. These sections were placed in a hybridizer, denatured at 85°C for 5 min, and hybridized at 37°C overnight. The slides were incubated in eluent for 5 min at 37°C, rinsed with 70% ethanol and dried naturally. After staining with 5 μl DAPI, the cells were observed under a fluorescence microscope.

The immunofluorescence value at each point in the tissue chip was calculated by Image-Pro Plus 6.0 software (Media Cybernetics, Inc., Rockville, MD, USA). The average optical density (AO) = cumulative optical density value (IOD)/pixel area of tissue (AREA). A higher AO value indicated a higher positive expression level.

### Quantitative real-time PCR

RNA from tissues and cells was isolated using TRIzol reagent (Invitrogen, USA) according to the manufacturer’s manual. The purified RNA was reverse synthesized into cDNA using the PrimeScript RT reagent Kit (Takara Biotech, Dalian, China). Then, we used SYBR Green Real-Time qPCR analysis (Roche Applied Science, Penzberg, Upper Bavaria, Germany) to analyze the transcriptional cDNA.

The sequences of the primers were as follows (5’-3’): lncRNA 604-F, AAAGAGAGCAAGAGGAGATCAAATC and lncRNA 604-R, CCAGGTCTCCACCCTTATGG (GUANGZHOU RIBOBIO CO., LTD, China); AEG-1-F, CGGAGCGAGGAACAGAAGAAGAAG and AEG-1-R, AACCAGAATCAGTCAGCACCTTATCAC; NF-κB-F, GGTGGACTACCTGGTGCCTCTAG and NF-κB-R, CGCCTCTGTCATTCGTGCTTCC; ERCC1-F, CTGCTGCTGCTGCTGCTTCC and ERCC1-R, GCTCCCACATCCACCAAGAAGAAG; GAPDH-F, ACGGATTTGGTCGTATTGGG and GAPDH-R, CGCTCCTGGAAGATGGTGAT (Sangon Biotechnology Inc., Shanghai, China). The relative expression level of transcripts was normalized to that of the internal control GAPDH and analyzed by using the 2^-ΔΔCt method.

### Western blot

Using the BCA method to determine the protein concentration, 100 µg protein was added to 7.5% SDS-PAGE for analysis. Then, the protein was transferred to a PVDF membrane after electrophoresis, blocked in TBST solution containing 5% skimmed milk powder, and incubated with antibody. The protocols were performed as previously described (18). The monoclonal rabbit anti-ZNF326 (1:1000, ABclonal Technology, USA, A16477), monoclonal rabbit anti-E-cadherin (1:1000, Cell Signaling Technology California, USA, #3195), monoclonal rabbit anti-N-cadherin (1:1000, Cell Signaling Technology California, USA, #13116), monoclonal rabbit anti-Vimentin (1:1000, Cell Signaling Technology California, USA, #5741), monoclonal rabbit anti-AEG-1 (1:1000, Abcam, England, ab227981), monoclonal rabbit anti- NF-κB (1:1000, Cell Signaling Technology California, USA, #8242), monoclonal rabbit anti-ERCC1 (1:1000, Abcam, England, ab129267), and monoclonal mouse anti-β-actin antibody (1:2000; Beyotime Biotechnology, Nantong, China, AF5001) were used as primary antibodies. ImageJ software (version 1.44, Wayne Rasband, National Institutes of Health, USA) was applied to quantify the correction of these protein bands with the corresponding β-actin level. The fold change of figures was relative to β-actin.

### Double luciferase reporter assay

The cells were divided into 24-well culture plates the day before plasmid transfection. We cloned the targeted and mutant sequences of lncRNA 604 and AEG-1 into the pmirGLO vector. According to the experimental design group, plasmid transfection experiments were performed by Lipofectamine 2000 (Invitrogen). After 24 h of transfection, the expression of fluorescent marker genes in the cells was observed under a fluorescence microscope, and then the Dual-Luciferase^®^ Reporter Assay System (E1910, Promega) kit was used to process the cells and detect luciferase expression. All experimental data were from three independent experiments.

### RNA pull-down and mass spectrometry analysis

Based on the PINK1-AS gene sequence, PCR was performed with plasmid DNA as a template to obtain the full-length sequence containing the T7 promoter. LncRNA 604 sense and antisense primer sequence probes were obtained and labeled with biotin. Then, the labeled RNA probe was incubated with the protein extract to form an RNA-protein complex. This complex was incubated with Pierce Nucleic-Acid Compatible Streptavidin Magnetic Beads for 30 min, eluted with biotin elution buffer, added to 5x SDS PAGE loading buffer, and transferred to ice immediately after boiling. Subsequently, we used SDS-PAGE for electrophoresis, stopped the electrophoresis 1 cm from the separation gel and cut off the gel. The gel was analyzed by mass spectrometry.

### *In vivo* assays

In the tumor xenograft model, approximately 2 × 10^6^ HT29 stable cells and negative control cells (0.2 ml/mouse; 5 mice/group) were implanted subcutaneously into the flanks of each mouse. The tumors were photographed each week, and after 21 days, the mice were sacrificed.

For the metastasis experiments, HT29 stable cells were injected into the abdominal cavity and tail veins of the mice (n = 5). In the abdominal metastasis model, HT29 stable cells were set as 0.2 ml/2 × 10^5^/mouse. The peritoneal metastases were observed and photographed each week. In the tail vein metastasis model, we adjusted the concentration of HT29 stable cells to 2 × 10^4^/ml and then administered 0.2 ml cell suspension into the tail veins. After 40 days, the metastases were photographed. At each time point of observation, 150 mg/kg D-luciferin (Gold Biotech, USA) was injected into the abdominal cavity of nude mice for 10 min, and then photographs were taken by a small-animal living imaging system (PerkinElmer, USA).

All animal experiments were conducted in accordance with the Committee of YangZhou University for the Use and Care of Animals.

### Statistical analysis

All data were collected and analyzed using SPSS (version 21.0) and STATA (version 10.1) software. The basic experimental data are the mean ± standard error (SD) of three independent experiments. All markers in tumor tissues and adjacent tissues were evaluated by the Wilcoxon test. Kaplan-Meier survival analysis was used to draw the survival curve. We used the Cox regression model to estimate the HRs and 95% CIs. P <0.05 was considered statistically significant.

## Results

### LncRNA 604 is elevated in CRC tissues and cell lines

**Figure 1:**
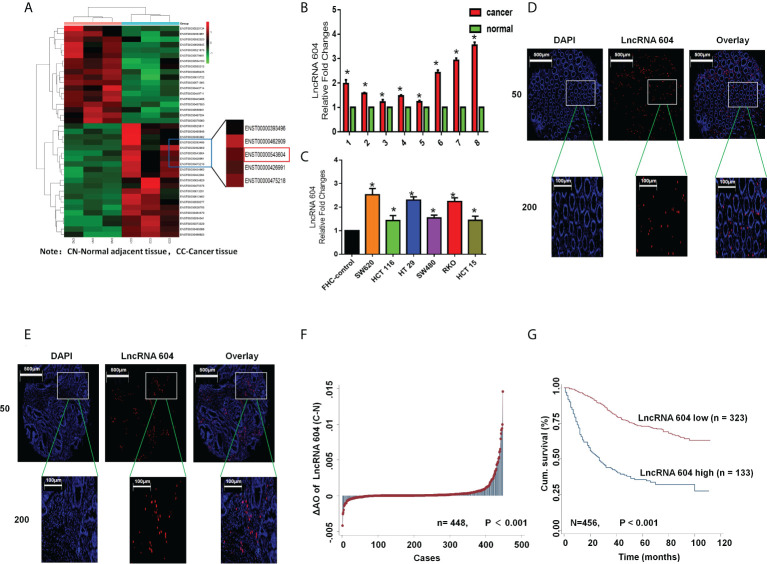
LncRNA 604 was consistently high in CRC tissues and cells. **(A)** LncRNA 604 was consistently higher in CRC tissues than in paracancerous tissues by high-throughput sequencing. **(B)** LncRNA 604 was high-expressed in CRC tissues compared with no-tumor tissues by qRT-PCR. **(C)** LncRNA 604 in CRC cells was significantly higher than that in FHC. **(D, E)** LncRNA 604 on the tissue microarrays was detected by FISH **(D)**: normal tissues; **(E)** cancer tissues. **(F)** LncRNA 604 in CRC tissues were significantly increased compared with normal tissues on the tissue microarrays. **(G)** The OS of CRC patients with low-expressed lncRNA 604 was significantly prolonged than patients with high-expressed lncRNA 604. All *P < 0.05

To study the expression of lncRNA 604 in CRC tissues and cells, fresh CRC tissues and corresponding paracancerous tissues were used for high-throughput sequencing. The paired fresh samples were frozen in liquid nitrogen immediately after surgical removal and maintained at −80°C until use for further experimental research. As shown in **Figure 1A**, we found that the expression of lncRNA 604 was consistently higher in CRC tissues than in paracancerous tissues. Then, we used qRT-PCR to detect the expression of lncRNA 604 in CRC tissues and cells. The results showed that lncRNA 604 was highly expressed in CRC tissues compared with paracancerous tissues (**Figure 1B**). The expression of lncRNA 604 in CRC cells (SW620, HCT116, HT29, SW480, RKO and HCT15) was significantly higher than that in FHC (**Figure 1C**).

To further verify the expression of lncRNA 604 in CRC, we constructed tissue microarrays of 470 CRC patients. The pathological data of each patient in the database are detailed in **Table S1**. We used FISH to detect the expression of lncRNA 604 on the tissue microarrays. Fluorescent images of cancer tissues and normal tissues were selected as shown in **Figures 1D, E**. We used Image-Pro Plus 6.0 software to calculate the specific value of each fluorescent spot in the tissue. Through the data analysis, we concluded that the expression of lncRNA 604 in CRC tissues was significantly increased compared with that in normal tissues (**Figure 1F**, P<0.001). Using Kaplan-Meier survival analysis, we found that the overall survival (OS) of CRC patients with low expression of lncRNA 604 was significantly prolonged compared with patients with high expression of lncRNA 604 (**Figure 1G**, P<0.001).

### LncRNA 604 promotes CRC cell proliferation and metastasis

**Figure 2:**
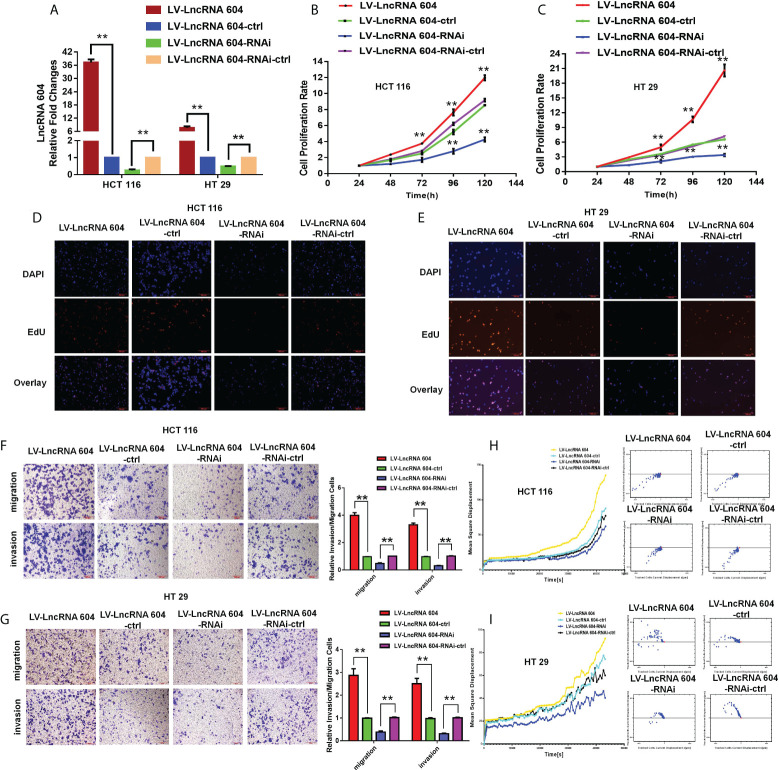
LncRNA 604 promoted CRC cells proliferation and metastasis *in vitro.* **(A)** The effect of virus interference for lncRNA 604, the experimental cells were divided into four groups: LV-LncRNA 604; LV-LncRNA 604-ctrl; LV-LncRNA 604-RNAi; LV-LncRNA 604-RNAi-ctrl. **(B, C)** LncRNA 604 could promote CRC cells proliferation by CCK8 assay [**(B)** HCT 116; C: HT 29]. **(D, E)** LncRNA 604 could promote CRC cells proliferation by EDU assay [**(D)** HCT 116; **(E)** HT 29]. **(F, H)** LncRNA 604 could enhance CRC cells invasion and migration by transwell assay [**(F)** HCT 116; **(H)** HT 29]. **(G, I)** LncRNA 604 could enhance CRC cells invasion and migration by high-content imaging system analysis [**(G)** HCT 116; **(I)** HT 29]. All **P < 0.05.

To explore the function of lncRNA 604 in CRC cells, we first constructed lentiviruses to vary the expression of lncRNA 604 in CRC cells. As shown in **Figure 2A**, overexpression of lncRNA 604 in HCT116 and HT29 cells was achieved using LV-LncRNA 604; LV-LncRNA 604-RNAi was used to knock down lncRNA 604 in cells. Then, we performed a CCK-8 assay to detect the proliferation of CRC cells when lncRNA 604 expression was altered. We found that overexpression of lncRNA 604 in HCT116 and HT29 cells promoted cell proliferation, while decreased expression had the opposite effect (**Figures 2B, C**; all P<0.05). We also performed an EdU assay and found that the proliferation capacity of CRC cells was elevated when lncRNA 604 was increased in HCT116 and HT29 cells, while the opposite was found when lncRNA 604 expression was decreased (**Figures 2D, E**).

To examine the function of lncRNA 604 in metastasis, we used Transwell assays and high-content imaging system analysis *in vitro*. Through these data, we concluded that the invasion and migration abilities of HCT116 and HT29 cells were significantly enhanced in LV-LncRNA 604 cells but weakened in LV-LncRNA 604-RNAi cells compared with the corresponding control group (**Figures 2F, G**, ** P < 0.01). As shown in **Figures 2H, I**, high-content imaging system analysis showed that the metastatic abilities of LV-LncRNA 604 cells were also elevated compared with those of the control group, but LV-LncRNA 604-RNAi cells showed the opposite trend.

### LncRNA 604 functions as a ceRNA to bind miRNA 564 and promotes tumor progression *via* the miRNA 564/AEG-1 signaling axis

**Figure 3:**
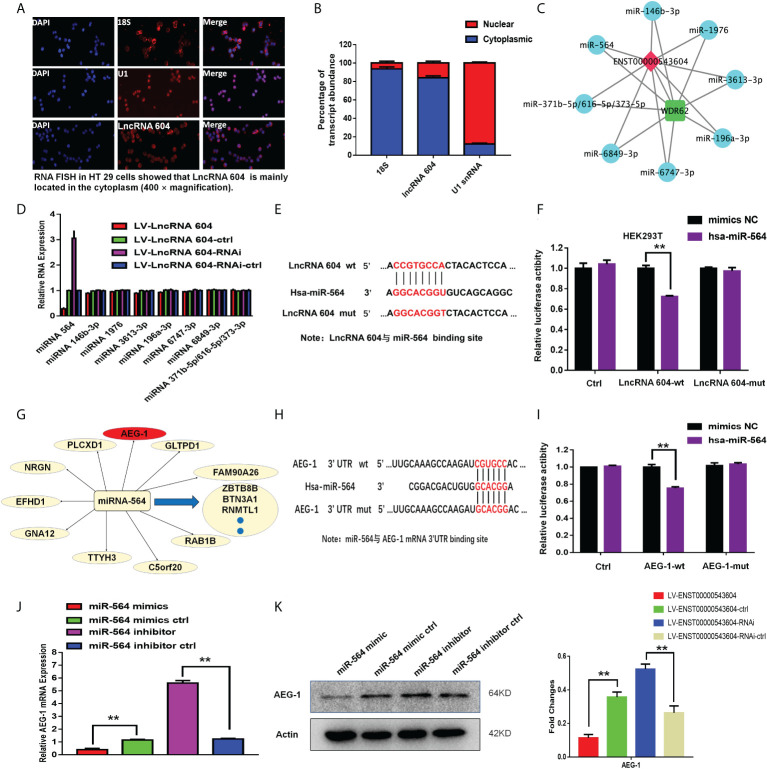
The mechanism of lncRNA 604 was detected as ceRNA. **(A)** The localization of lncRNA 604 was detected by FISH assay in the cells. **(B)** The expression of lncRNA 604 compared with the nuclear localization U1 and the cytoplasmic localization marker 18S. **(C)** LncRNA 604 spatial signal regulation network was generated by Targetscan software. **(D)** MiRNA 564 was regulated by lncRNA 604 by qRT-PCR in these bound miRNAs. **(E)** The binding site of lncRNA 604 and miRNA 564. **(F)** MiRNA 564 bounded the wild type of lncRNA 604 by the dual luciferin reporter gene. **(G)** The putative target genes of miRNA 564 was predicted by MiRanda (http://www.microrna.org). **(H)** The binding site of miRNA 564 and AEG-1. **(I)** miRNA 564 mimics reduced the luciferase activity of wt 3’UTR of AEG-1, while had no effect on mut 3’TUR of AEG-1. **(J, K)** MiRNA 564 could negatively regulate AEG-1 by qRT-PCR and WB [**(J)** RNA; **(K)** protein]. All **P < 0.05.

To study the mechanism of action of lncRNA 604, we first used a FISH assay to detect its expression and localization in the cells. The results showed that lncRNA 604 was mostly located in the cytoplasm, and only a small part was expressed in the nucleus compared with the nuclear localization marker U1 and the cytoplasmic localization marker 18S (**Figures 3A, B**). Then, we used TargetScan software to predict the lncRNA 604 spatial signal regulation network. As shown in **Figure 3C**, we found that numerous miRNAs bound to lncRNA 604, such as miRNA 564, miRNA 146b-3p, and miRNA 1976.

From these bound miRNAs, qRT-PCR was used to verify whether a regulatory relationship existed. We found that miRNA 564 was regulated by lncRNA 604 (**Figure 3D**). Subsequently, we used a dual luciferin reporter assay to verify this result. As shown in **Figure 3E**, we predicted the binding site of lncRNA 604 and miRNA 564 through bioinformatics analysis. The wild-type lncRNA 604 luciferase plasmid containing miRNA 564 binding sites and a mutant lncRNA 604 plasmid were generated. These plasmids were cotransfected with miRNA into HEK293T cells. We found that miRNA 564 could reduce wild-type lncRNA 604 luciferase activity, but mutant lncRNA 604 activity was not changed (**Figure 3F**). The results indicated that miRNA 564 bound to the wild-type lncRNA 604.

We used MiRanda (http://www.microrna.org) to search for putative target genes of miRNA 564 (**Figure 3G**). The 3’-UTR of AEG-1 was predicted to be the putative binding site of miRNA 564 because it contained a region matching the seed sequence of miRNA 564 (**Figure 3H**). Accordingly, the dual luciferin reporter assay was used for further validation. As shown in **Figure 3I**, miRNA 564 mimics reduced the luciferase activity of the wt 3’UTR of AEG-1 but had no effect on the luciferase activity of the mut 3’UTR of AEG-1. Through qRT-PCR and WB assays, we confirmed that miRNA 564 could negatively regulate AEG-1 at both the RNA and protein levels (**Figures 3J, K**). Taken together, we concluded that the lncRNA 604/miR-564/AEG-1 axis was important in CRC and that lncRNA 604 could promote tumor progression by regulating miRNA 564 and AEG-1.

### LncRNA 604 binds nuclear transcription factor ZNF326

**Figure 4:**
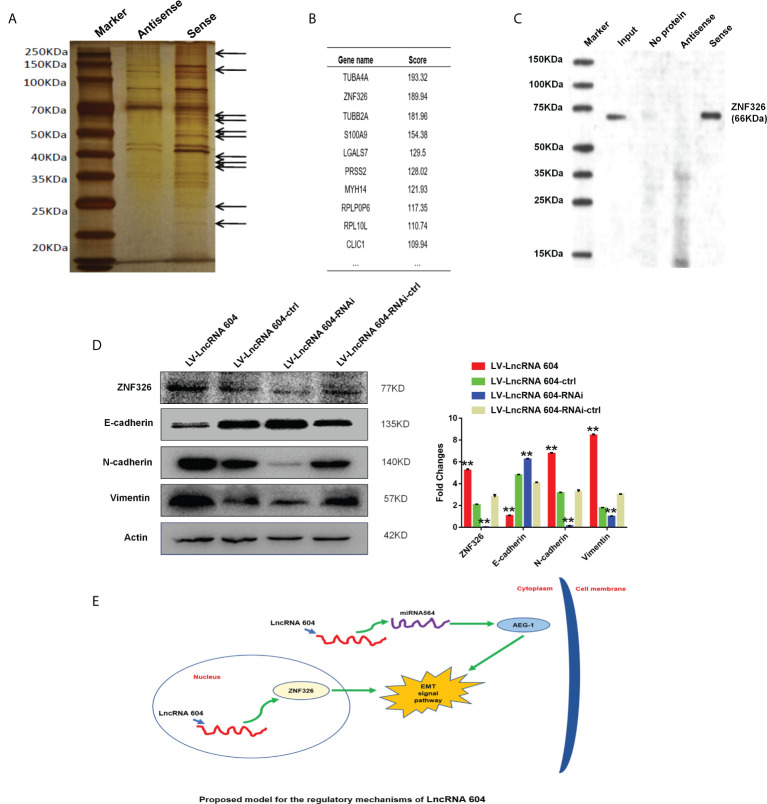
The mechanism of lncRNA 604 was detected as binding nuclear transcription factor. **(A)** The silver staining indicated that all bound proteins were pulled out by RNA pull down assay. **(B)** ZNF326 was the second most differential protein in lncRNA 604-binding product by mass spectrometry. **(C)** ZNF326 expression was confirmed this specific binding with lncRNA 604 by WB. **(D)** LncRNA 604 could positively regulate ZNF326, N-cad, Vim expression and decrease E-cad expression. **(E)** The totle mechanism signal route: lncRNA 604 could inhibit AEG-1 by combining with miRNA564 in the cytoplasm and regulate the nuclear transcription factor ZNF326 in nucleus, which could promote the occurrence of EMT and lead to CRC metastasis. All **P < 0.05.

Through the results of the FISH experiment, we found that lncRNA 604 was mostly located in the cytoplasm, but a small portion existed in the nucleus. Previously, we found that lncRNA 604 could adsorb miRNA564 in the cytoplasm through a ceRNA mechanism, thereby promoting tumor metastasis. To further study whether lncRNA 604 in the nucleus plays a biological function, we used an RNA pulldown assay to pull out all bound proteins by silver staining (**Figure 4A**). Subsequently, all of the proteins were identified by mass spectrometry. As shown in **Figure 4B**, ZNF326 was the second most differentially expressed protein in the lncRNA 604-binding product. We also performed WB to confirm this specific binding (**Figure 4C**).

ZNF326 is a nuclear transcription factor and acts as an oncoprotein in breast cancer, promoting the occurrence of EMT. We used the WB assay to detect important proteins of the EMT signaling pathway. The results showed that ZNF326, N-cad, and Vim expression was increased, and E-cad expression was decreased in the lncRNA 604 high expression group; ZNF326, N-cad, and Vim expression was decreased, and the expression of E-cad was increased in the lncRNA 604 low expression group compared with the corresponding control group (**Figure 4D**).

According to the above mechanistic research results, we concluded that lncRNA 604 may not only inhibit AEG-1 by combining with miRNA564 in the cytoplasm but also regulate the nuclear transcription factor ZNF326 in the nucleus, which could promote the occurrence of EMT and lead to CRC metastasis (**Figure 4E**).

### LncRNA 604 accelerates CRC cell growth and metastasis *in vivo*

**Figure 5:**
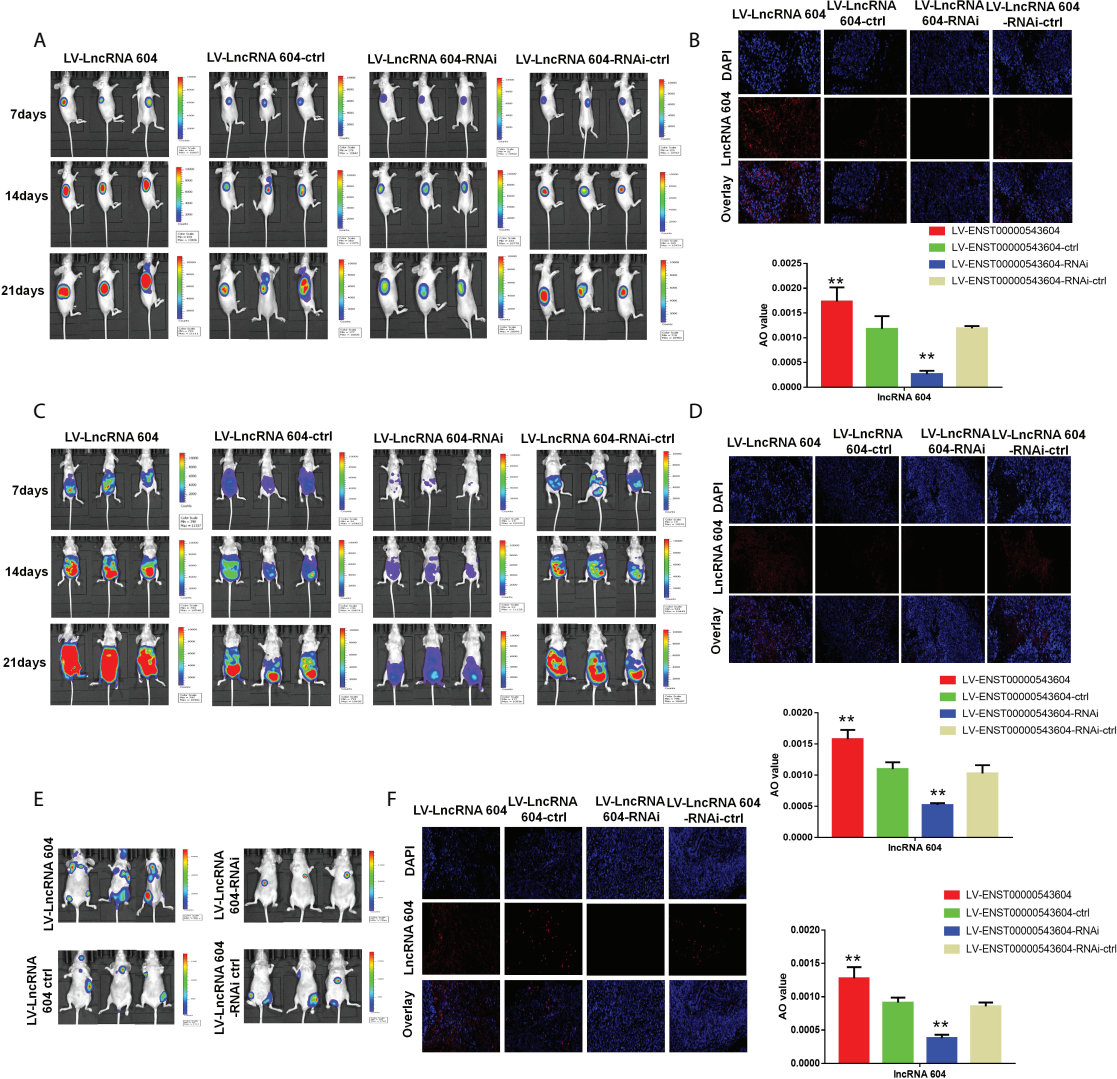
LncRNA 604 promoted CRC cell growth and metastasis *in vivo.* **(A)** The growth of subcutaneous tumor was observed in every group by a small-animal living imaging system: tumour growth increased in LV-lncRNA 604 group, however suppressed in LV-lncRNA 604-RNAi group. **(B)** The expression of lncRNA 604 in the xenograft tumors was detected by FISH (magnification 50×). **(C)** Much more metastases were found in LV-lncRNA 604 group, however the LV-lncRNA 604-RNAi group was opposite in abdominal cavity metastasis model. **(D)** The lncRNA 604 expression of abdominal metastases was consistent with cells (magnification 50×). **(E)** In the tail vein metastasis model, there were more multiple metastases in LV-lncRNA 604 group, however LV-lncRNA 604-RNAi group was significantly less than respective control group. **(F)** The lncRNA 604 expression of all metastases was detected by FISH (magnification 50×). All **P < 0.05.

To investigate the functional role of lncRNA 604 in CRC cell proliferation *in vivo*, luciferase-expressing LV-lncRNA 604 or LV-lncRNA 604-RNAi HCT116 cells were implanted subcutaneously into the flanks of nude mice. At days 7, 14 and 21, we injected fluorescein potassium into the abdominal cavity of nude mice and then observed the size of tumors using a small-animal living imaging system after 15 min. Tumor growth was dramatically increased in the LV-lncRNA 604 group but suppressed in the LV-lncRNA 604-RNAi group compared with the corresponding control group (**Figure 5A**). Simultaneously, we detected the expression of lncRNA 604 in xenograft tumors by FISH. The trend of lncRNA 604 expression in tissues was consistent with that in cells (**Figure 5B**, all P < 0.01).

In addition, LV-lncRNA 604 or LV-lncRNA 604-RNAi HCT116 cells were injected into the peritoneal cavity of BALB/c nude mice. We observed abdominal metastases at days 7, 14 and 21. As shown in **Figure 5C**, more metastases were found in the LV-lncRNA 604 group than in the control group; however, the LV-lncRNA 604-RNAi group showed the opposite result. The lncRNA 604 expression in abdominal metastases was consistent with that in cells (**Figure 5D**, all P < 0.01). Furthermore, we used the tail vein metastasis model to study the role of lncRNA 604 in tumor metastasis. At day 40, we found that there were more metastases in the LV-lncRNA 604 group than in the control group, but the LV-lncRNA 604-RNAi group had significantly fewer metastases than the control group (**Figure 5E**). As expected, lncRNA 604 expression in metastases was consistent with that in cells (**Figure 5F**, all P < 0.01). From these results, we concluded that lncRNA 604 could promote CRC cell growth and metastasis *in vivo*.

### LncRNA 604 alone or in combination with its regulatory molecules predicts the prognosis of CRC

**Figure 6:**
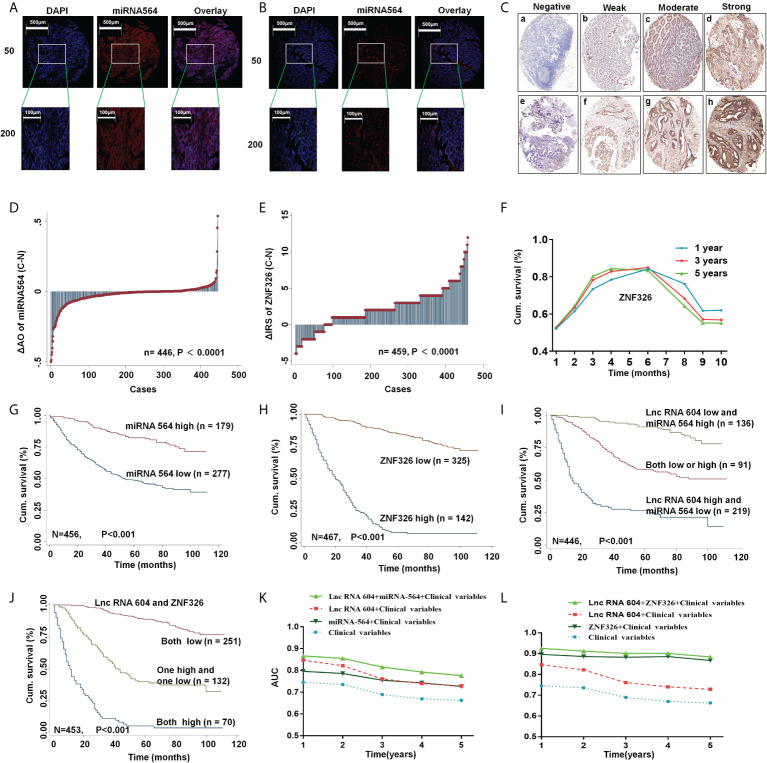
LncRNA 604 with its regulatory molecules predicted the prognosis of CRC. **(A, B)** The typical pictures of miRNA 564 was detected by FISH (A)cancer; (B) normal tissue). **(C)** The typical pictures of ZNF326 was detected by IHC. **(D, E)** Molecular expression was analyzed in tumor and normal tissues (**D**) miRNA 564; **(E)** ZNF326). **(F)** The cutoff value of ZNF326 was calculated by ROC analysis. **(G, H)** High miRNA 564 or low ZNF326 expression had a better OS in CRC patients by Kaplan-Meier survival analysis. [**(G)** miRNA 564; **(H)** ZNF326]. **(I, J)** LncRNA 604 combined with its regulatory molecules had the best survival prognosis by Kaplan-Meier survival analysis [**(I)** lncRNA 604 and miRNA 564; **(J)** lncRNA 604 and ZNF326]. **(K, L)** ROC analysis indicated lncRNA 604 combined with miRNA 564 or ZNF326 had a synergistic effect on prognosis of CRC patient [**(K)** lncRNA 604 and miRNA 564; **(L)** lncRNA 604 and ZNF326].

To further explore the clinical value of lncRNA 604, we used Fisher’s exact analysis to analyze the correlation between lncRNA 604, miRNA 564, ZNF326 and patients’ clinicopathological parameters in the CRC cohort. We performed FISH assays and immunohistochemistry to detect miRNA 564 and ZNF326, respectively. The same method as lncRNA 604 was used to obtain the expressed value of miRNA 564, and the expression of ZNF326 was assessed according to our previously published paper (17). Representative photos are shown in **Figures 6A–C**. As shown in **Table 1**, lncRNA 604 expression had a significant positive association with lymph node metastasis, TNM stage and distant metastasis (all P < 0.001); miRNA 564 expression was significantly associated with depth of invasion, lymph node metastasis, TNM stage (all P < 0.001) and distant metastasis (P < 0.05); ZNF326 expression was significantly associated with age (P < 0.05), pathological classification, depth of invasion, lymph node metastasis, and TNM stage (all P < 0.001). **Figure 6D** shows that miRNA 564 expression was lower in tumor tissues than in nontumor tissues (P < 0.001). Furthermore, we found that ZNF326 expression was higher in tumor tissues than in nontumor tissues (P < 0.001; **Figure 6E**).

**Table 1:** Relationship between expression levels of Lnc RNA 604, miRNA 564, ZNF326 and clinicopathological features in CRC patients.

Variables	Lnc RNA 604			miRNA 564			ZNF 326		
		n = 456 cases			n = 456 cases			n = 467 cases		
		low (%)	high (%)	P^a^	low (%)	high (%)	P^a^	low (%)	high (%)	P^a^
All patients	323 (70.83)	133 (29.17)		277 (60.7)	179 (39.3)		325 (69.6)	142 (30.4)	
Age (years)			0.414			0.378			0.004
≤65	185 (71.4)	74 (28.6)		154 (59.9)	103 (40.1)		198 (74.7)	67 (25.3)	
>65	138 (70.1)	59 (29.9)		123 (61.8)	76 (38.2)		127 (62.9)	75 (37.1)	
Gender			0.085			0.120			0.528
Males	199 (73.4)	72 (26.6)		156 (57.8)	114 (42.2)		195 (69.6)	85 (30.4)	
Females	124 (67.0)	61 (33.0)		121 (65.1)	65 (34.9)		130 (69.5)	57 (30.5)	
Pathological classification^b^			0.445			0.104			<0.001
I	4 (80.0)	1 (20.0)		1 (25.0)	3 (75.0)		4 (80.0)	1 (20.0)	
II	292 (71.4)	117 (28.6)		244 (59.5)	166 (40.5)		303 (72.0)	118 (28.0)	
III	22 (47.8)	24 (52.2)		26 (72.2)	10 (27.8)		14 (38.9)	22 (61.1)	
Depth of invasion^b^			0.076			<0.001			<0.001
T1+T2	76 (76.8)	23 (23.2)		43 (43.9)	55 (56.1)		93 (84.3)	9 (15.7)	
T3+T4	242 (68.8)	110 (31.2)		230 (65.2)	123 (34.8)		228 (56.0)	133 (44.0)	
Lymph node metastasis^b^			<0.001			<0.001			<0.001
N0	214 (80.5)	52 (19.5)		118 (44.7)	146 (55.3)		216 (78.8)	58 (21.2)	
N1+N2+N3	105 (56.5)	81 (43.5)		156 (83.0)	32 (17.0)		106 (55.8)	84 (44.2)	
TNM stage^b^			<0.001			<0.001			<0.001
I	66 (78.6)	18 (21.4)		34 (40.5)	50 (59.5)		80 (92.0)	7 (8.0)	
II	143 (82.7)	30 (17.3)		78 (45.6)	93 (54.4)		134 (75.3)	44 (24.7)	
III	73 (50.0)	73 (50.0)		147 (82.6)	31 (17.4)		101 (56.1)	79 (43.9)	
IV	12 (50.0)	12 (50.0)		14 (82.4)	3 (17.6)		5 (29.4)	12 (70.6)	
Tumor diameter^b^			0.093			0.530			0.114
≤5 cm	266 (72.3)	102 (27.7)		222 (60.8)	143 (39.2)		266 (70.9)	109 (29.1)	
>5 cm	56 (64.4)	31 (35.6)		55 (61.1)	35 (38.9)		58 (65.9)	33 (34.1)	
Distant metastasis			<0.001			0.024			<0.001
M0	318 (72.8)	119 (27.2)		261 (59.7)	176 (40.3)		320 (71.4)	128 (28.6)	
M1	5 (26.3)	14 (73.7)		16 (84.2)	3 (15.8)		5 (26.3)	14 (73.7)	

a Two-sided Fisher’s exact tests.

b Some patients missing these clinical pathological parameters.

The cutoff values of lncRNA 604 and miRNA 564 were calculated by Cutoff Finder (http://molpath.charite.de/cutoff/index.jsp). We used receiver operator characteristic (ROC) analysis to obtain the cutoff value of the semiquantitative immunoreactivity score (IRS) for ZNF326, as reported elsewhere (19). **Figure 6F** indicates that IRS 0-4 and IRS 6-12 were classified as low or high expression of ZNF326, respectively. Through Kaplan-Meier survival analysis, we found that CRC patients with high miRNA 564 or low ZNF326 expression had a better OS (all P < 0.001; **Figures 6G, H**). As shown in **Table 2** and **Table 3**, lncRNA 604, miRNA 564 or ZNF326 was an independent marker for the prognosis of CRC patients by univariate and multivariate Cox regression analysis.

**Table 2:** Univariate Cox regression analysis of Lnc RNA 604, miRNA 564, ZNF326 expression and clinicopathological variables predicting survival in CRC patients.

Variables	n = 470 cases	
		HR (95 % CI)	P
Age (≤65 vs. > 65)	1.607 (1.215-2.126)	0.001
Gender (male vs. female)	1.013 (0.762-1.347)	0.927
Pathological classification (I/II vs. III)	2.475 (1.587-3.860)	<0.001
Depth of invasion (T1/T2 vs. T3/T4)	3.687 (2.270-5.990)	<0.001
Lymph node metastasis (N0 vs. N1/N2)	2.807 (2.112-3.731)	<0.001
TNM stage (I/II vs. III/IV)	3.214 (2.407-4.291)	<0.001
Distant metastasis (M0 vs. M1)	8.150 (4.849-13.699)	<0.001
Tumor diameter (≤5 cm vs. >5 cm)	1.196 (0.848-1.688)	0.307
Lnc RNA 604 expression (low vs. high)	0.279 (0.209-0.372)	<0.001
miRNA 564 expression (low vs. high)	0.289 (0.204-0.411)	<0.001
ZNF 326 expression (low vs. high)	0.075 (0.055-0.103)	<0.001

**Table 3:** Multivariate Cox regression analysis of Lnc RNA 604, miRNA564, ZNF326, Lnc RNA 604/miRNA564, Lnc RNA 604/ZNF 326 expression and clinicopathological variables predicting survival in patients with CRC.

Variables	HR (95% CI)	P^a^
**Lnc RNA 604**		
Age (≤65 vs. > 65)	1.868 (1.398-2.496)	<0.001
Gender (male vs. female)	0.797 (0.593-1.071)	0.132
Pathological classification (I/II vs. III)	2.133 (1.318-3.451)	0.002
TNM stage (I/II vs. III/IV)	2.943 (2.167-3.998)	<0.001
Tumor diameter (≤5 cm vs. >5 cm)	1.065 (0.731-1.552)	0.743
Lnc RNA 604 expression (low vs. high)	0.372 (0.250-0.554)	<0.001
**miRNA564**		
Age (≤65 vs. > 65)	1.862 (1.394-2.487)	<0.001
Gender (male vs. female)	0.833 (0.623-1.116)	0.221
Pathological classification (I/II vs. III)	2.063 (1.291-3.296)	0.002
TNM stage (I/II vs. III/IV)	2.584 (1.889-3.533)	<0.001
Tumor diameter (≤5 cm vs. >5 cm)	1.057 (0.733-1.525)	0.767
miRNA564 expression (low vs. high)	0.396 (0.274-0.571)	<0.001
**ZNF326**		
Age (≤65 vs. > 65)	1.495 (1.116-2.003)	0.007
Gender (male vs. female)	0.969 (0.727-1.292)	0.834
Pathological classification (I/II vs. III)	1.723 (1.082-2.744)	0.022
TNM stage (I/II vs. III/IV)	2.198 (1.625-2.972)	<0.001
Tumor diameter (≤5 cm vs. >5 cm)	1.118 (0.778-1.606)	0.548
ZNF 326 expression (low vs. high)	0.096 (0.069-0.133)	<0.001
**Lnc RNA 604/miRNA564**		
Age (≤65 vs. > 65)	1.858 (1.386-2.489)	<0.001
Gender (male vs. female)	0.868 (0.647-1.163)	0.634
Pathological classification (I/II vs. III)	2.116 (1.317-3.398)	0.002
TNM stage (I/II vs. III/IV)	2.944 (2.165-4.005)	<0.001
Tumor diameter (≤5 cm vs. >5 cm)	1.039 (0.714-1.512)	0.840
Lnc RNA 604/miRNA564 expression		
Lnc RNA 604 low and miRNA564 high vs. both low or high	0.499 (0.354-0.705)	<0.001
Lnc RNA 604 low and miRNA564 high vs. Lnc RNA 604 high and miRNA564 low	0.271 (0.167-0.438)	<0.001
**Lnc RNA 604/ZNF326**		
Age (≤65 vs. > 65)	1.904 (1.432-2.530)	<0.001
Gender (male vs. female)	0.902 (0.677-1.203)	0.483
Pathological classification (I/II vs. III)	1.958 (1.232-3.111)	0.004
TNM stage (I/II vs. III/IV)	3.357 (2.500-4.508)	<0.001
Tumor diameter (≤5 cm vs. >5 cm)	1.155 (0.807-1.652)	0.430
Lnc RNA 604/ZNF326 expression		
Both low vs. both high	0.038 (0.023-0.062)	<0.001
Both low vs.one high and one low	5.215 (3.563-7.632)	<0.001

a Multivariate Cox regression analysis including gender, pathological classification, TNMstage, tumor diameter, Lnc RNA 604, miRNA564, ZNF326, Lnc RNA 604/miRNA564, Lnc RNA 604/ZNF326 expression status.

According to the above mechanistic research, we concluded that lncRNA 604 promoted CRC cell growth and metastasis through miRNA 564 or ZNF326. These three indicators were all effective molecular markers for predicting the prognosis of CRC patients. We hypothesized whether lncRNA 604 could be combined with other markers to better predict the prognosis of CRC patients. We used the Kaplan-Meier survival analysis method again to investigate this hypothesis. The results showed that patients with low expression of lncRNA 604 and high expression of miRNA 564 had the best survival prognosis compared with both the high/low expression group or the lncRNA 604 high and miRNA 564 low expression group (P < 0.001; **Figure 6I**). As shown in **Figure 6J**, we also noted that patients with low expression of lncRNA 604 and ZNF326 had a more favorable survival outcome than both the high expression group and the high and low expression groups (P < 0.001). Simultaneously, multivariate Cox regression analysis indicated that low expression of lncRNA 604 and high expression of miRNA 564 and low expression of lncRNA 604 and ZNF326 were favorable independent prognostic factors (all P < 0.001, **Table 3**). In addition, we conducted a time-dependent ROC analysis to further evaluate whether lncRNA 604 combined with miRNA 564 or ZNF326 had a synergistic effect on the prognosis of CRC patients. The clinical risk scores (TNM stage, histologic type, and tumor diameter), lncRNA 604 or miRNA 564 or lncRNA 604 plus miRNA 564 combined with the clinical risk scores are shown in **Figure 6K**. Our data indicated that lncRNA 604 plus miRNA 564 combined with the clinical risk scores contributed more than any one of these markers alone. As shown in **Figure 6L**, our data also indicated that lncRNA 604 plus ZNF326 combined with the clinical risk scores had the best predictive effect on the prognosis of CRC compared with the other three groups.

### LncRNA 604 promotes CRC cell chemoresistance

**Figure 7:**
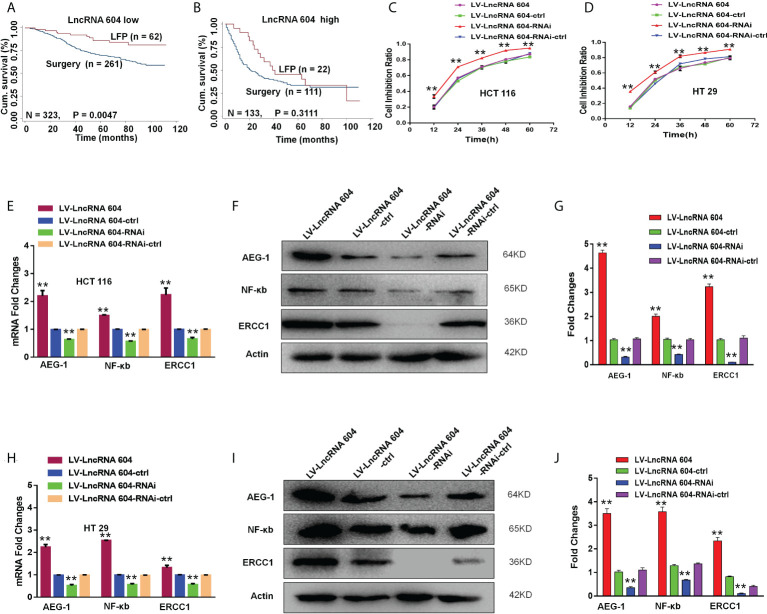
LncRNA 604 promoted CRC cell chemoresistance by improving the expression of AEG-1, NF-κb, and ERCC1. **(A, B)** CRC patients with low-expressed or high-expressed lncRNA 604 who adopted postoperative LFP regimen compared with patients who received surgery alone on OS by Kaplan-Meier curve method **(A)** low-expressed lncRNA 604; **(B)** high-expressed lncRNA 604). **(C, D)** LV-lncRNA 604-RNAi CRC cells were more sensitive than control group, however LV-lncRNA 604 cells were not sensitive by CCK8 assay *in vitro* **(C)** HCT 116; **(D)** HT 29). **(E–G)** AEG-1, NF-κb, and ERCC1 in HCT 116 cells were changed with the expression of lncRNA 604 by RT-PCR and WB **(E)** RNA; **(F)**, **(G)** protein). **(H–J)** AEG-1, NF-κb, and ERCC1 in HCT 29 cells were changed with the expression of lncRNA 604 by RT-PCR and WB **(H)** RNA; **(I)**, **(J)** protein. All **P < 0.05.

We analyzed the database using the Kaplan-Meier curve method and found that patients with low lncRNA 604 expression had a significantly longer survival time from the postoperative LFP regimen (5-Fu+L-OHP) than patients who received surgery alone (P < 0.05, **Figure 7A**), while patients with high lncRNA 604 expression did not benefit (P>0.05, **Figure 7B**). To further investigate this finding, we performed a CCK-8 assay to detect cell proliferation when LV-lncRNA 604 or LV-lncRNA 604-RNAi CRC cells were in the presence of 20 μg/ml 5-Fu and 20 μg/ml L-OHP. Our data indicated that LV-lncRNA 604-RNAi CRC cells were more sensitive than the control group; however, LV-lncRNA 604 cells were not sensitive (**Figure 7C**: HCT116; **Figure 7D**: HT29).

**Figure 8:**
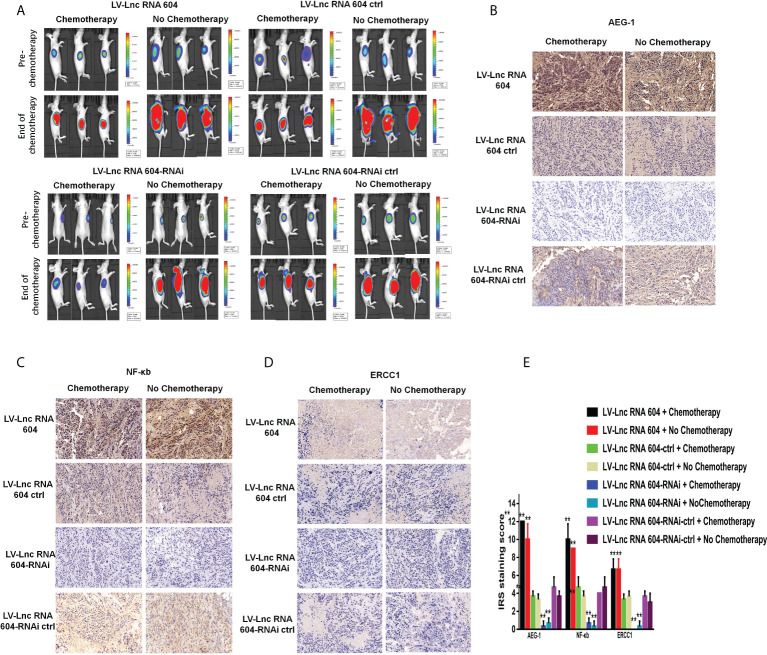
LncRNA 604 promoted CRC cell chemoresistance *in vivo*. **(A)** The subcutaneous tumors were significantly smaller in low-expressed lncRNA 604 group while the high-expressed lncRNA 604 group failed to benefit, compared with their respective control groups. **(B–D)** AEG-1, NF-κb, and ERCC1 in subcutaneous tumor were detected by IHC, the trends were the same as WB *in vitro* **(B)** AEG-1; **(C)** NF-κb; **(D)** ERCC1). **(E)** The IRS staining score of AEG-1, NF-κb, and ERCC in all groups was graded. All **P < 0.05. The symbol **** represents the statistical significance of the two groups: black and red pillars.

To explore the corresponding mechanism, we used RT-PCR and WB to detect drug resistance proteins. We found that AEG-1, NF-κB, and ERCC1 in HCT116 cells changed with the expression of lncRNA 604 regardless of RNA (**Figure 7E**) or protein levels (**Figures 7F, G**). As shown in **Figures 7H–J**, the test result of HT29 was consistent with HCT116. Then, we performed animal experiments to verify this finding *in vivo*. The model of subcutaneous metastasis was the same as described previously. After 14 days, 25 mg/kg 5-Fu and 5 mg/kg L-OHP were injected into the abdominal cavity once a week three times. After one cycle of chemotherapy, the subcutaneous tumor was observed using a small-animal living imaging system. All the nude mice were sacrificed. These data showed that the tumors were significantly smaller in the low-expression lncRNA 604 group, while the high-expression lncRNA 604 group failed to benefit compared with their respective control groups (**Figure 8A**). We calculated the tumor inhibition rate according to the formula = (1-experimental group tumor weight/control group tumor weight)*100%. The tumor inhibition rate of each group is shown in **Table 4**. The results showed that the tumor inhibition rate of the low-expression lncRNA 604 group after chemotherapy was significantly different from that of the control group, but there was no difference in the high-expression group. Subsequently, the protein expression of AEG-1, NF-κB, and ERCC1 in subcutaneous tumors was detected using IHC. The results indicated that the expression levels of AEG-1, NF-κB, and ERCC1 were higher in the high-expression lncRNA 604 group than in the low-expression lncRNA 604 group (**Figures 8B–D**). As shown in **Figure 8E**, the IRS staining scores of all groups were determined. Based on these experimental results, we concluded that lncRNA 604 may increase the drug resistance of CRC cells by enhancing the expression of AEG-1, NF-κB, and ERCC1.

**Table 4:** Effects of chemotherapeutic drugs on subcutaneous tumor constructed by CRC cell lines of high-expressed or low-expressed lncRNA 604 and respective control cell ( X ± S, n = 3).

Group	Tumor weight (g)	Tumor inhibition rate (%)
LV-LncRNA 604	1.731±0.222	
LV-LncRNA 604+Chemotherapy	0.588±0.103	66.0%
LV-LncRNA 604-ctrl	1.097±0.074	
LV-LncRNA 604-ctrl+Chemotherapy	0.365±0.021	66.7%^△^
LV-LncRNA 604-RNAi	0.747±0.120	
LV-LncRNA 604-RNAi+Chemotherapy	0.191±0.046	74.4%
LV-LncRNA 604-RNAi-ctrl	0.945±0.121	
LV-LncRNA 604-RNAi-ctrl+Chemotherapy	0.337±0.125	64.4%^*^

Compared with the respective control group, ^*^P < 0.05, ^△^P > 0.05.

## Discussion

In recent years, lncRNAs have been shown to play an important role in the occurrence and development of many cancers (20, 21), including CRC (22, 23). In this article, we performed high-throughput sequencing on 3 cases of fresh CRC and corresponding adjacent tissues. The results showed that lncRNA 604 was highly expressed in CRC tissues. Then, we verified this conclusion again in cell, tissue and sample databases. However, whether lncRNA 604 has a biological function and the related mechanism of action had not been studied.

In our study, we attempted to identify the function and mechanism of lncRNA 604 in CRC. Through *in vivo* and *in vitro* experiments, our data indicated that lncRNA 604 promoted the proliferation and metastasis of CRC cells. To elucidate the possible mechanism of lncRNA 604, we used a FISH assay to show that lncRNA 604 was mostly located in the cytoplasm and that only a small portion was located in the nucleus. The common mechanism of lncRNAs, such as lncRNAs in the cytoplasm, has a ceRNA mode that binds miRNAs; lncRNAs in the nucleus can bind nuclear transcription factors. Considering these two mechanisms of action, we attempted to identify which mechanism is used by lncRNA 604. Research results showed that miRNA 564 could be directly combined with lncRNA 604. Many studies have shown that miRNA 564 has antitumor effects in cancer (24–26). In addition, we found that miRNA 564 had antitumor effects through negative regulation of AEG-1. Studies have confirmed that AEG-1 could promote the occurrence of EMT (27, 28). Therefore, we concluded that lncRNA 604 may promote EMT through the miRNA 564/AEG-1 signaling axis in the cytoplasm, which leads to CRC metastasis. We also used an RNA pulldown experiment to pull down all of the proteins that bind to lncRNA 604 and then performed mass spectrometry analysis. We found that lncRNA 604 binds to the nuclear transcription factor ZNF326 and could positively regulate its expression. Furthermore, we demonstrated that ZNF326 could accelerate the occurrence of EMT (29, 30). Therefore, we also concluded that lncRNA 604 could promote the metastasis of CRC by regulating ZNF326 in the nucleus.

Previous studies have shown that lncRNAs have been increasingly reported to be related to the prognosis of cancer patients (31–33). In the pathogenesis of CRC, an increasing number of lncRNAs that have important clinical value have been identified. LncRNAs, such as ZFAS1, SNHG11, LINC00909 and LINC00654, could be used as effective markers for predicting prognosis and early diagnosis of CRC (34, 35). In this study, lncRNA 604 expression was significantly higher in CRC tissues than in normal adjacent tissues. Simultaneously, we used the Kaplan-Meier survival method and Cox regression to analyze the relationship of lncRNA 604 and its regulatory molecules with the prognosis of CRC. Our data indicated that lncRNA 604, miRNA 564 and ZNF326 were all independent predictors of the prognosis of CRC. Notably, lncRNA 604 combined with miRNA 564 or ZNF326 had a synergistic effect, which was better than the prediction effect of any single molecule.

Chemotherapy drug resistance is the main reason for the failure of CRC treatment (36). Many lncRNAs are related to the sensitivity to chemotherapy drugs (36, 37). LncRNA CRNDE regulates the Wnt/β-catenin signaling pathway through miR-181a-5p, promoting the proliferation of CRC cells and increasing the tolerance to chemotherapy drugs (38). In the present article, we first found that low expression of lncRNA 604 in CRC patients with LFP after postoperative chemotherapy could significantly prolong OS, while high expression of lncRNA 604 in CRC patients did not benefit. Then, we verified this conclusion by drug sensitivity experiments *in vitro*. CRC cells expressing low levels of lncRNA 604 were more sensitive to chemotherapy drugs than control cells. Our data showed that lncRNA 604 could increase the expression of AEG-1, NF-κB, and ERCC1, resulting in drug resistance in CRC cells.

In our investigation, we studied the function, mechanism and clinical value of lncRNA 604 in CRC. We have demonstrated that the lncRNA 604/miRNA 564/AEG-1/EMT or lncRNA 604/ZNF326/EMT signaling axis may play an important role in CRC progression. LncRNA 604 is an independent and effective molecule for predicting the prognosis of CRC. We also established that lncRNA 604 could increase drug resistance protein expression, resulting in chemoresistance in CRC cells. Our study may provide new insights for CRC treatments.

## Data availability statement

The original contributions presented in the study are included in the article/**Supplementary Files**. Further inquiries can be directed to the corresponding authors.

## Ethics statement

Animal studies were performed in accordance with the National Institutes of Health Guide for the Care and Use of Laboratory Animals. Written informed consent was obtained from the owners for the participation of their animals in this study. Written informed consent was obtained from the individual(s) for the publication of any potentially identifiable images or data included in this article.

## Author contributions

WW and ZZ contributed to the conception and design of the study. WW, XD and HW contributed to the conception, design and editing of the manuscript. JJ and KM contributed to the statistical analysis. ZZ, YW and ML contributed to complete experiments. YL and YZ critically reviewed the manuscript. All authors contributed to the article and approved the submitted version.

## Funding

The work was financially supported by Top Talent Support Program for young and middle-aged people of Wuxi Health Committee (awarded to WW); Wuxi City Health Planning Commission project (No. MS201815, awarded to WW; No. Z201907, awarded to YZ); the Natural Science Foundation of Jiangsu Province (No. BK20191149, awarded to YZ).

## Acknowledgments

The authors want to thank all the participants in the research.

## Conflict of interest

The authors declare that the research was conducted in the absence of any commercial or financial relationships that could be construed as a potential conflict of interest.

## Publisher’s note

All claims expressed in this article are solely those of the authors and do not necessarily represent those of their affiliated organizations, or those of the publisher, the editors and the reviewers. Any product that may be evaluated in this article, or claim that may be made by its manufacturer, is not guaranteed or endorsed by the publisher.
