# Supplementary material for: LncRNA-ENST00000543604 exerts a tumor-promoting effect via miRNA 564/AEG-1 or ZNF326/EMT and predicts the prognosis of and chemotherapeutic effect in colorectal cancer
Source: Front Oncol. 2022 Aug 23;12:960481. doi: 10.3389/fonc.2022.960481 (PMC9445881; doi:10.3389/fonc.2022.960481)
Supplement: Supplementary file 1 [file Table_1.docx]

**Table S1**. the patients' clinicopathologic information in CRC

| Variables | n | |
| --- | --- | --- |
| All patients | 456 | (%) |
| Age (years) | | |
| ≤65 | 259 | 56.8 |
| ＞65 | 197 | 43.2 |
| Gender | | |
| Males | 271 | 59.43 |
| Females | 185 | 40.57 |
| Pathological classification^b^ | | |
| Ⅰ | 5 | 1.11 |
| Ⅱ | 409 | 90.89 |
| Ⅲ | 36 | 8.0 |
| Depth of invasion^b^ | | |
| T1+ T2 | 99 | 21.95 |
| T3+ T4 | 352 | 78.05 |
| Lymph node metastasis^b^ | | |
| N0 | 266 | 58.85 |
| N1+ N2+N3 | 186 | 41.15 |
| TNM stage^b^ | | |
| Ⅰ | 84 | 18.67 |
| Ⅱ | 173 | 38.44 |
| Ⅲ | 176 | 39.11 |
| Ⅳ | 17 | 3.78 |
| Tumor diameter^b^ | | |
| ≤5 cm | 368 | 80.88 |
| ＞5 cm | 87 | 19.12 |
| Distant metastasis | | |
| M0 | 437 | 95.83 |
| M1 | 19 | 4.17 |

^b^ Some patients missing these clinical pathological parameters
